# In vitro assessment of the pathogenicity of the LDLR c.2160delC variant in familial hypercholesterolemia

**DOI:** 10.1186/s12944-023-01848-6

**Published:** 2023-06-20

**Authors:** Shaoyi Lin, Tingting Hu, Kaihan Wang, Jiaqi Wang, Yunyun Zhu, Xiaomin Chen

**Affiliations:** 1 Department of Cardiology, The First Affiliated Hospital of Ningbo University, Ningbo, Zhejiang China; 2 Zhejiang University School of Medicine, Hangzhou, Zhejiang China; 3 Department of Geriatrics, The First Affiliated Hospital of Ningbo University, Ningbo, Zhejiang China

**Keywords:** Familial hypercholesterolemia, Low-density lipoprotein cholesterol, Low-density lipoprotein receptor, Whole-exome sequencing, Endoplasmic reticulum

## Abstract

**Background:**

Familial hypercholesterolemia (FH) is an inherited disorder with markedly elevated low-density lipoprotein cholesterol (LDL-C) and premature atherosclerotic cardiovascular disease. Although many mutations have been reported in FH, only a few have been identified as pathogenic mutations. This study aimed to confirm the pathogenicity of the LDL receptor (LDLR) c.2160delC variant in FH.

**Methods:**

In this study, the proband and her family members were systematically investigated, and a pedigree map was drawn. High-throughput whole-exome sequencing was used to explore the variants in this family. Next, quantitative polymerase chain reaction (qPCR), western blot (WB) assays, and flow cytometry were conducted to detect the effect of the LDLR c.2160delC variant on its expression. The LDL uptake capacity and cell localization of LDLR variants were analyzed by confocal microscopy.

**Results:**

According to Dutch Lipid Clinic Network (DLCN) diagnostic criteria, three FH patients were identified with the LDLR c.2160delC variant in this family. An in-silico analysis suggested that the deletion mutation at the 2160 site of LDLR causes a termination mutation. The results of qPCR and WB verified that the LDLR c.2160delC variant led to early termination of LDLR gene transcription. Furthermore, the LDLR c.2160delC variant caused LDLR to accumulate in the endoplasmic reticulum, preventing it from reaching the cell surface and internalizing LDL.

**Conclusions:**

The LDLR c.2160delC variant is a terminating mutation that plays a pathogenic role in FH.

**Supplementary Information:**

The online version contains supplementary material available at 10.1186/s12944-023-01848-6.

## Introduction

Familial hypercholesterolemia (FH) is a prevalent hereditary metabolic disorder featuring tendinous or cutaneous xanthomas, extremely low-density lipoprotein cholesterol (LDL-C), arcus corneal, and early onset of cardiovascular disease [1–3]. Current evidence suggests that FH has a prevalence of 1 in 311 among the general population and is more common among atherosclerotic cardiovascular disease (ASCVD) cases [4–6]. Although significant inroads have been achieved over the past few years, FH remains underdiagnosed and undertreated worldwide [3, 7]. Therefore, strengthening population screening and cascade screening of probands are necessary and effective means to improve the diagnosis rate.

However, treating FH remains a major conundrum for clinicians. Statins, which form the basis of cholesterol-lowering therapy, exhibit significant variations in their ability to lower LDL-C among individuals and have many adverse effects [6]. Most importantly, the therapeutic effect of statins is below par for FH patients [8]. PCSK9 inhibitors represent a new class of drugs for treating this patient population. In the latest CREDIT-2 study, tafolecimab was related to a significant decrease in LDL-C levels in Chinese heterozygous FH patients (-57.4% for 150 mg Q2 W; -61.9% for 450 mg Q4 W) [9]. However, there is an increasing consensus that the lipid-lowering effect of PCSK9 inhibitors for homozygous FH patients is not ideal in adults and children [10–12]. In addition to drugs, lipoprotein apheresis has been identified to reduce LDL-C and Lp(a), and long-term maintenance of lipoprotein apheresis can effectively delay atherosclerosis and promote xanthomata regression in patients with FH [13]. Liver transplantation is a highly effective therapeutic method for introducing functional LDLR, which can rapidly reduce LDL-C to the normal range and inhibit the occurrence and progression of the disease [14, 15]. However, the long-term risk-benefit ratio of patients with FH undergoing lipoprotein apheresis or liver transplantation remains to be evaluated due to several complications and liver source tension. All these findings urge us to promptly investigate the pathogenesis of FH and conduct further research for more effective therapies to resolve this conundrum.

It has been established that FH typically arises from genetic mutations that play a vital role in regulating cholesterol balance [16]. LDLR, APOB, PCSK9, and LDLRAP1 are the causative genes identified thus far, with the LDL receptor (LDLR) being the most commonly mutated [1, 17]. More than 4000 LDLR variants have been identified according to the UCL database (http://www.lovd.nl/LDLR) [18, 19]. As a transmembrane receptor mainly expressed in the liver, LDLR can bind to circulating LDL particles, subsequently internalizing and degrading LDL [12]. An increasing body of literature suggests that mutations at different locations may cause LDLR failure through different mechanisms, such as impaired binding to LDL, impaired endocytosis, and impaired transport [20–22]. Even two LDLR variants encoding the same amino acid impair LDLR function differently [23]. Based on these findings, the functionality of the newly identified variants warrants a thorough investigation, providing the theoretical basis for exploring effective FH treatment strategies.

In this study, our objective was to enhance the mutation spectrum of FH-related genes to support gene screening in high-risk populations with FH. Additionally, we conducted cell biology investigations to determine the pathogenicity of the newly identified LDLR variant, LDLR c.2160delC, in individuals with FH.

## Methods and materials

### Study population and sample collection

The proband and her family members were recruited from the First Affiliated Hospital of Ningbo University. Six participants were included in the study. All subjects underwent a detailed physical examination, and the pedigree was drawn. FH diagnosis was mainly based on the Dutch Lipid Clinic Network (DLCN) criteria. In addition, blood samples were obtained from all participants for subsequent studies. This study was approved by the Ethics Committee of the First Affiliated Hospital of Ningbo University (2019-R020), and written informed consent for participation and publication was signed by all subjects.

### Whole-exome sequencing

An Omega Blood DNA Kit (Omega Bio-Tek, Georgia State, USA) was used to isolate genomic DNA from blood samples. A NanoDrop 2000 spectrophotometer (Thermo Fisher Scientific, Waltham, USA) was used to detect the quality of extracted DNA. Whole-exome sequencing was completed by BGI (Shenzhen, China). First, the original data were processed to generate high-quality clean data, which were then combined with a reference genome (GRCh37/hg19) to obtain the original alignment results in the bam file. Finally, small fragment insertion/deletion and single nucleotide variants were recognized.

### Sanger sequencing

Validation of the next-generation sequencing (NGS) results was performed by Sanger sequencing. The primers for LDLR were designed on Primer 5.0 (F: 5’-GTCATCTTCCTTGCTGCCTGTTTAG-3’, R: 5’-GTTTCCACAAGGAGGTTTCAAGGT-3’). PCRs were performed in 50 µL of a mixture containing 25 µL 2XES Taq Master Mix (CW0690H, CWBIO, Jiangsu, China), 2 µL primer, 500 ng DNA, and enzyme-free water (supplemented to 50 µL). Amplification was performed on Mastercycler® nexus X2 (Eppendorf, Hamburg, Germany) with the following parameters: 94℃ 2 min; 40 cycles: 94℃ 30 s, 59℃ 30 s, 72℃ 30 s; 72 2 min. The amplified products were sent to BGI for Sanger sequencing, and Chromas software was applied to analyze the sequencing results. Sequencing results were compared with the reference genome (GRCh37/hg19) to confirm the mutations in this family.

### In silico analysis

The pathogenic potential of gene variants was analyzed by MutationTaster 2021 (http://www.mutationtaster.org/) [24]. MutationTaster 2021 uses all intragenic variants from the 1000 Genomes Project, ExAC, or gnomAD in which there is at least one homozygous carrier as benign training cases, and the deleterious training cases comprise intragenic disease mutations from the Professional Version of the Human Gene Mutation Database (HGMD® Pro) and from ClinVar [25]. Based on the above training datasets, the random forest model was constructed as a classifier that can predict the effect of a specific DNA variant. For LDLR, the reference genome NM_000527.4 was used.

### Generation of mutant expression constructs

Plasmids were synthesized by GENECHEM (Shanghai, China). All variants described in this study were based on the encoded LDLR sequence (NM_000527.4). GFP was added to the vector to assess the efficiency of plasmid transfection in living cells. Site-directed mutagenesis was applied to introduce the 2160delC mutation into the LDLR expression vector. A blank plasmid carrying only the GFP sequence was used as a negative control, and a plasmid carrying both the normal LDLR-encoding sequence (WT) and GFP sequence was used as a positive control. The experimental group was transfected with both mutant LDLR and GFP plasmids. Each experiment was repeated three times.

### Cell culture and transfection

Human embryonic kidney cells (HEK-293T) were cultured in DMEM (Cytiva, Shanghai, China) supplemented with 100 U/mL penicillin/streptomycin (Beyotime, Shanghai, China) and 10% FBS (Pan-Biotech, Adenbach, Germany). After HEK-293T cells reached 80% confluence, plasmid transfection was performed using Lipofectamine 2000 Reagent (Invitrogen, Carlsbad, USA).

### Quantitative real-time PCR

Forty-eight hours after transfection, RNA-Solv® Reagent (Omega, Connecticut, USA) was used for total RNA extraction. Reverse transcription was performed using the HiScript cDNA Synthesis Kit (CW2569M, CWBIO, Jiangsu, China), which was performed on Mastercycler® nexus X2 (Eppendorf, Hamburg, Germany) with the following parameters: 42℃ for 50 min and 85℃ for 5 min. This study was conducted using FastStart Essential DNA Green Master Mix (069242204001, Roche, Basel, Switzerland) and LightCycler®480 (Roche, Basel, Switzerland) for quantitative real-time PCR. The primers for LDLR were F: 5’-CCTGACTCCGCTTCTTCTGCCCCAG-3’ and R: 5’-ACGCAGAAACAAGGCGTGTGCCAC-3’. The primers for GAPDH were F: 5’-GGAGTCAACGGATTTGGT-3’ and R: 5’-GTGATGGGATTTCCATTGAT-3’. GAPDH was chosen as the internal reference, and each test was carried out in triplicate. LDLR variant expression was measured using the 2^−ΔΔCt^ method.

### Western blotting

RAPI buffer (Solarbio, Beijing, China) containing protease and phosphatase inhibitors was used to lyse the samples. The cellular lysates were added to 1× loading buffer (Beyotime, Shanghai, China), which was run on an 8% SDS‒PAGE gradient gel at 120 V for 90 min. Then, the proteins were transferred onto PVDF membranes (Merck, Darmstadt, Germany). After blocking, the primary antibodies monoclonal mouse anti-LDLR (1:1000, ab204941, Abcam, Cambridge, UK) and monoclonal rabbit anti-β-actin (1:5000, AC026, ABclonal, Wuhan, China) were used in this study. The proteins were visualized using a fully automated chemiluminescence/fluorescence image analysis system (Tanon, Shanghai, China), and the protein integration density was quantified using ImageJ software.

### Flow cytometry

After transfection, cells were collected into 1.5 mL EP tubes (0.5-1 × 10^6^ per tube). Then, HEK-293T cells were blocked and cultured with anti-human LDLR monoclonal antibody conjugated with APC (5 µL/test, ab275614, Abcam, Cambridge, UK) for 30 min at RT. Fluorescence intensity was measured on a Beckman CytoFlex S flow cytometer (Beckman Coultern, California, USA) using the filter FL3 (excitation: 645 nm, emission: 660 nm).

### Immunofluorescence staining

For immunostaining, HEK-293T cells were fixed for 10 min in 4% paraformaldehyde. Cells were blocked in 5% donkey serum diluted with 0.1% TBST and then incubated with mouse anti-LDLR antibody (1:800, ab204941, Abcam, Cambridge, UK) and rabbit anti-calnexin antibody (1:100, P27824, CST, Danvers, USA) for 12 h at 4 °C. After incubation with the respective secondary antibodies, confocal microscopy was used to determine whether the protein was trapped in the endoplasmic reticulum.

Additionally, the uptake capacity of LDLR variants was analyzed by immunocytochemistry. After a confluence of 50% was reached, the medium was changed to DMEM with 0.3% FBS. Twelve hours later, the cells were incubated at 37 °C for an additional 4 h with 20 µg/mL Dil-LDL (Thermo Fisher Scientific, Waltham, USA). After 4% paraformaldehyde fixation, HEK-293T cells on coverslips were stained with DAPI and visualized with confocal microscopy.

### Statistical analyses

The statistical analyses were carried out using SPSS 25.0 software (SPSS Inc., Chicago, USA). Continuous variables with normal distribution are shown as the mean ± SD and were compared by t test. A *P* value < 0.05 in the two-tailed analysis was considered statistically significant. The experimental results were plotted with GraphPad Prism 8 software (GraphPad Software, La Jolla, CA).

## Results

### Clinical characteristics of the proband and her family

**Fig. 1:**
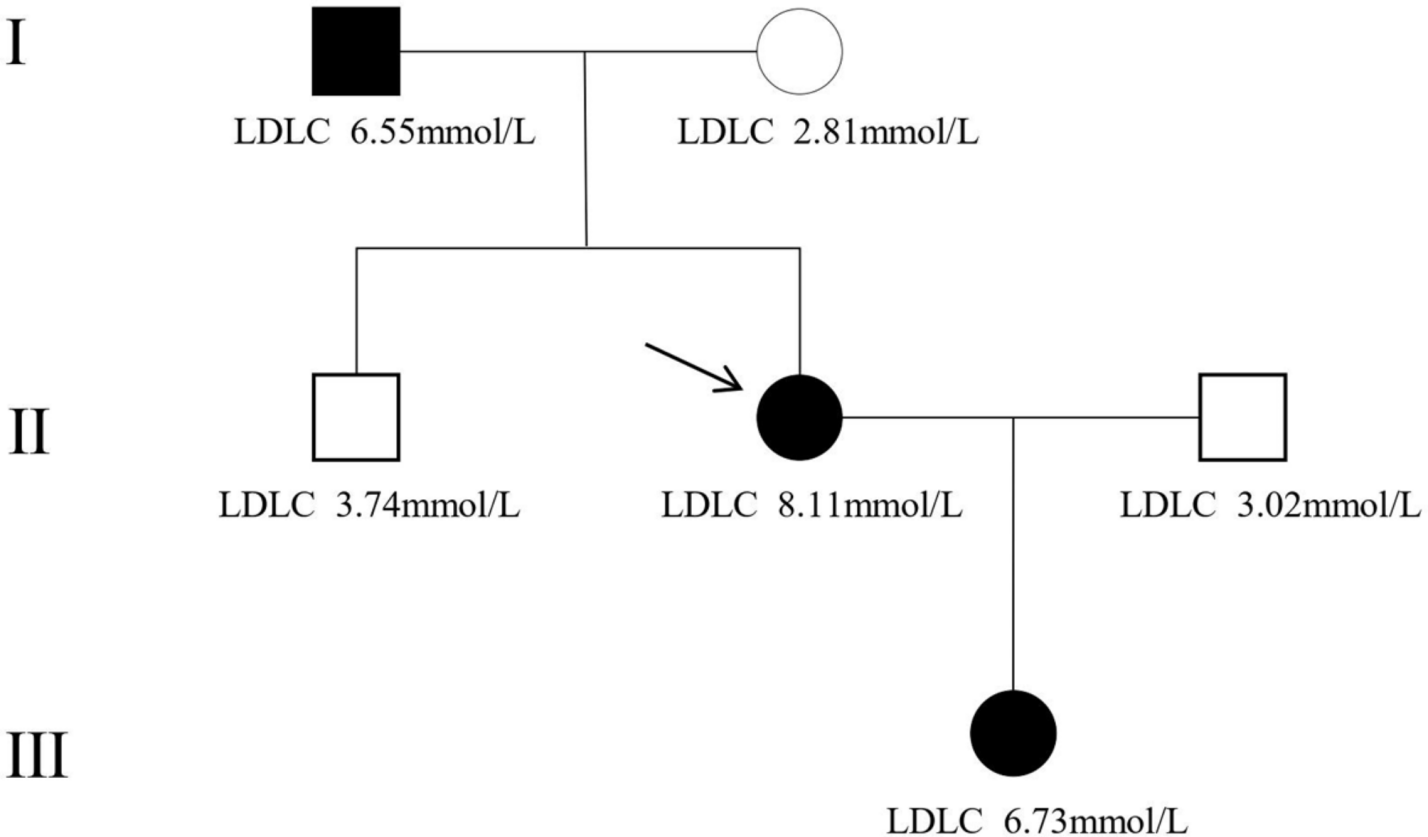
Family tree of the proband. The arrow indicates the proband

The proband was a 33-year-old female enrolled at The First Affiliated Hospital of Ningbo University for dyslipidemia and corneal arch. At baseline, the total cholesterol level was 11.26 mmol/L, and the LDL-C level was 8.11 mmol/L. The patient’s family history revealed that her father, brother, and daughter also had dyslipidemia (Fig. 1). According to the DLCN diagnostic criteria, the proband and her father and daughter were suspected of having FH (Table 1).

**Table 1:** The DLCN scores of participants in this family

Dutch Lipid Clinic Network Criteria		Participants scores					
Criteria	Score	I-1	I-2	II-1	II-2	II-3	III-1
Family History							
First-degree relative with premature coronary and/or vascular disease (men ≤ 55 years, women ≤ 60 years), OR	1	2	2	2	2	2	2
First-degree relative with known LDL-cholesterol ≥ 95th percentile for age and sex	1						
First-degree relative with tendon xanthomata and/or arcus cornelis, OR	2						
Children aged ≤ 18 years with known LDL-cholesterol ≥ 95th percentile for age and sex	2						
**Clinical History**							
Patient with premature coronary artery disease (age as above)	2	1	0	0	2	0	0
Patient with premature cerebral or peripheral vascular disease (age as above)	1						
**Physical Examination**							
Tendon Xanthomas	6	4	0	0	4	0	0
Arcus Cornelis at age ≤ 45 years	4						
**LDL Cholesterol (mmol/L) (mg/dL)**							
LDL-C ≥ 8.5 (330)	8						
LDL-C 6.5–8.4 (250–329)	5	5	0	0	5	0	5
LDL-C 5.0-6.4 (190–249)	3						
LDL-C 4.0-4.9 (155–189)	1						
**DNA Analysis** – functional mutation LDLR, APOB and PCSK9	8	/	/	/	8	0	8
**Total Score**		12	2	2	21	2	15

/means unknown. The highest score was awarded to each group

### Pathogenic variants and in-silico analysis

**Fig. 2:**
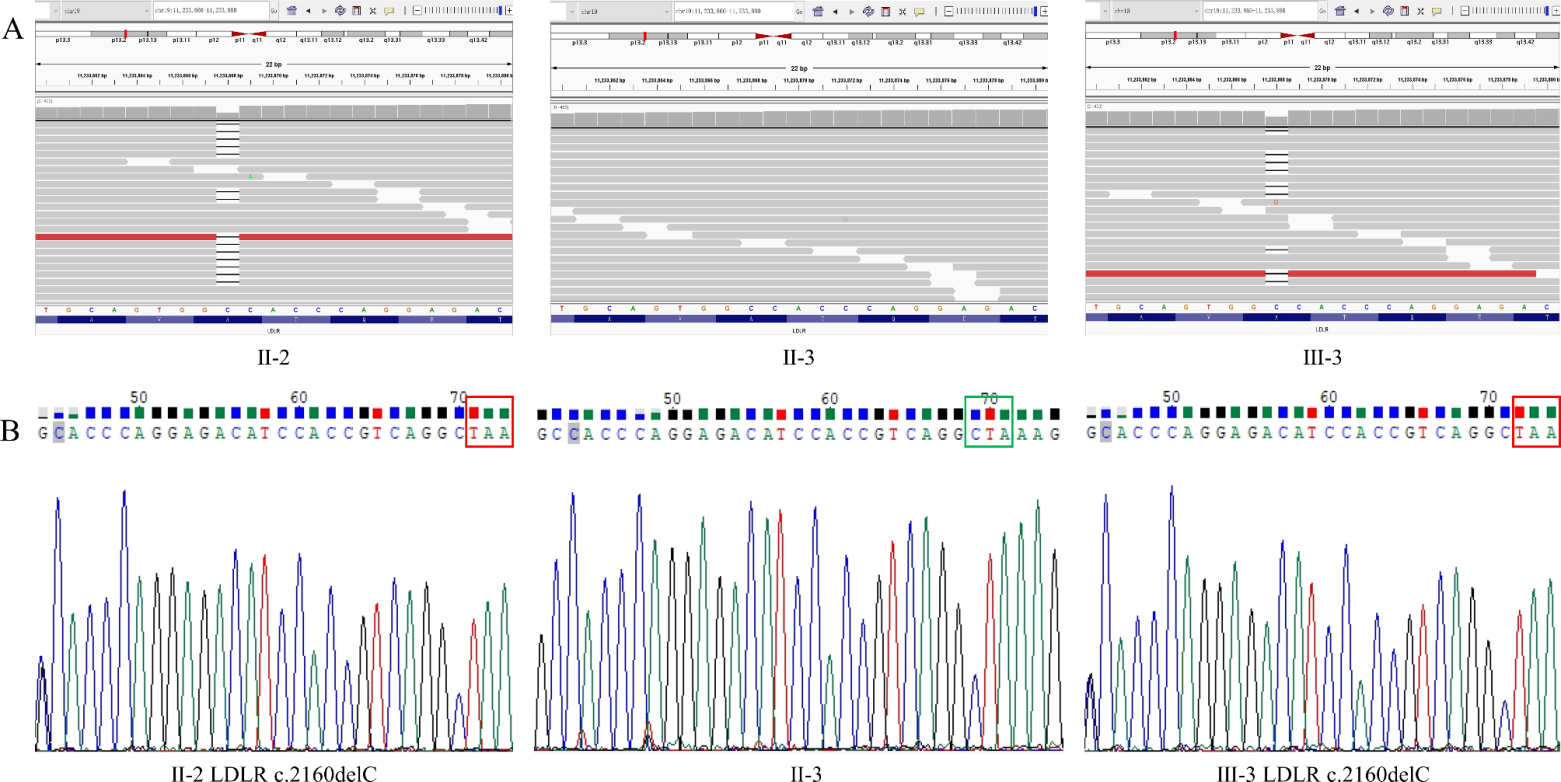
Pathogenic variants and in silico analysis. **(A)** The results of whole-exome sequencing. **(B)** Sanger sequencing confirmed the existence of LDLR c.2160delC. The reverse primer was used for Sanger sequencing. The shaded area represents the actual base. The contents of the box are the stop codon induced by the deletion mutation of the 2160 locus

Through whole-exon sequencing (II-2, II-3, III-1) and bioinformatics analysis, a potential pathogenic mutation was identified in this family, located at the 2160 site of LDLR (Fig. 2A). Sanger sequencing further confirmed the existence of LDLR c.2160delC in the corresponding patients (Fig. 2B). MutationTaster2021 software was used to predict the pathogenicity of the novel LDLR variant LDLR c.2160delC in FH. LDLR c.2160delC variant represented a potential pathogenic variant, which could lead to early termination of mRNA transcription.

### LDLR c.2160delC variant affected the expression of LDLR

**Fig. 3:**
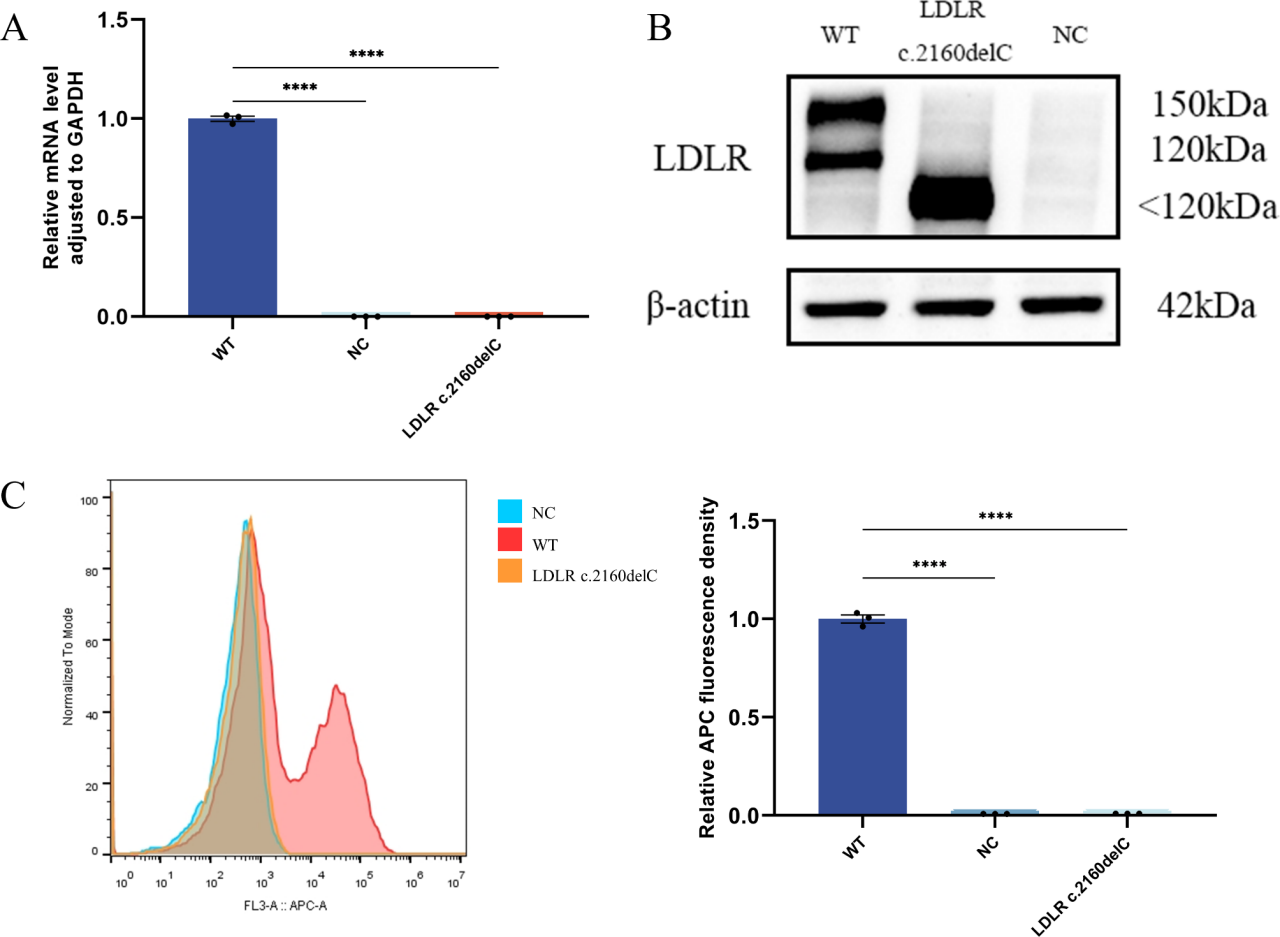
LDLR c.2160delC variant affected the expression of LDLR. **(A)** The mRNA expression level of LDLR c.2160delC in HEK293T cells. **(B)** The protein expression of LDLR c.2160delC in HEK293T cells. **(C)** LDLR expression on the HEK293T cell surface. **** represents *P* < 0.01

Next, qPCR, WB, and flow cytometry assays were conducted to validate that LDLR c.2160delC could induce premature transcription termination. Based on the mutation site 2160 of the gene sequence, PCR primers were designed, and LDLR mRNA expression levels were extremely low compared to WT (Fig. 3A, *P* < 0.01). LDLR protein expression in each group was analyzed by western blotting (Fig. 3B). In the group expressing WT LDLR, the precursor form is about 120 kDa, and the mature form is about 150 kDa. Since LDLR c.2160delC caused premature termination, the protein’s size was smaller than normal LDLR (< 120 kDa). Moreover, neither precursors nor mature forms were observed in the variant group. In addition, LDLR on the cell surface was further analyzed by flow cytometry (Fig. 3C). There was almost no LDLR on the surface in the LDLR c.2160delC group. These results indicated that LDLR c.2160delC was a terminating mutation that could prevent LDLR transport to the cell membrane.

### LDLR c.2160delC variant accumulated in the endoplasmic reticulum and reduced Dil-LDL uptake in HEK-293T cells

**Fig. 4:**
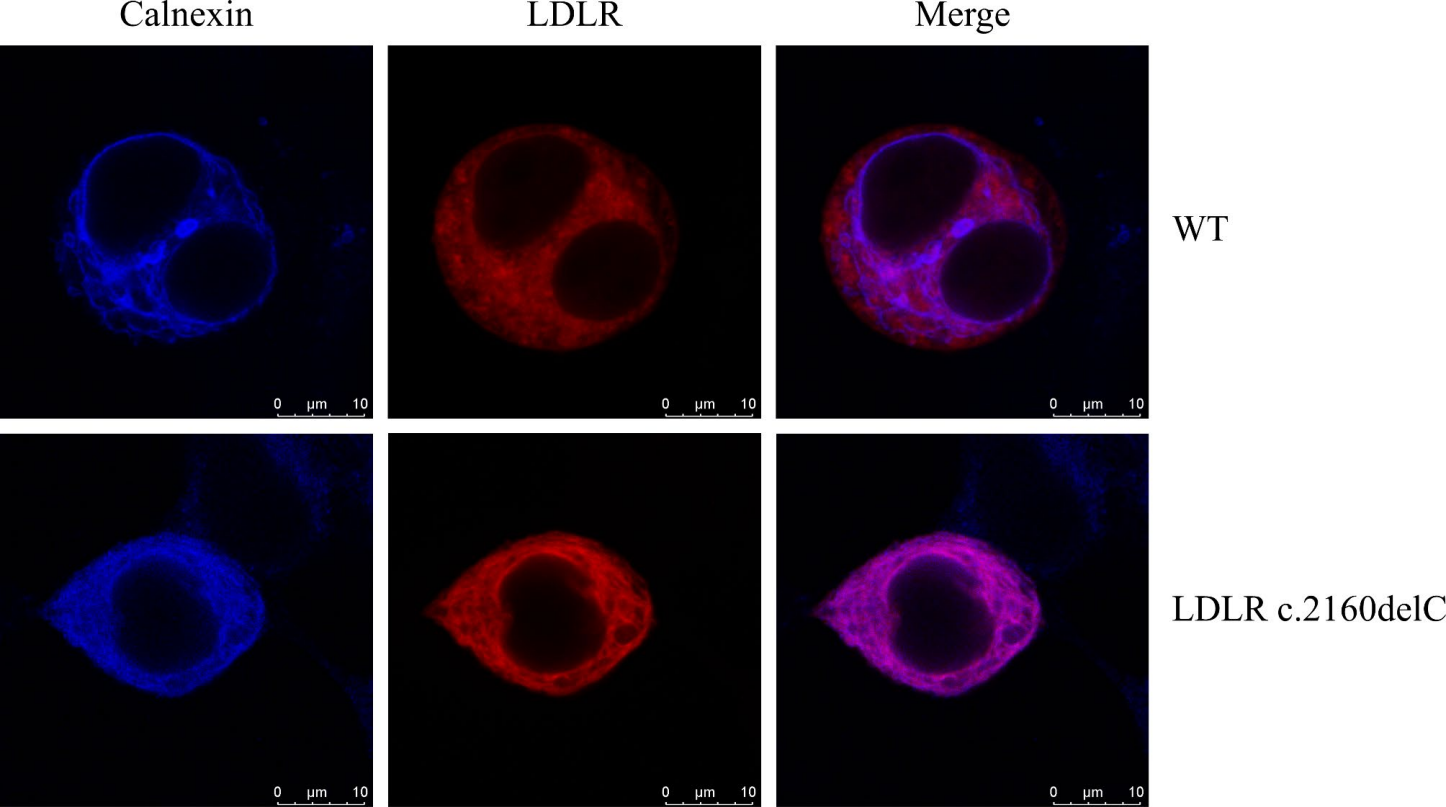
Representative confocal microscopy images of the localization of LDLR in the endoplasmic reticulum. Blue fluorescence represents the endoplasmic reticulum, while red fluorescence represents LDLR

**Fig. 5:**
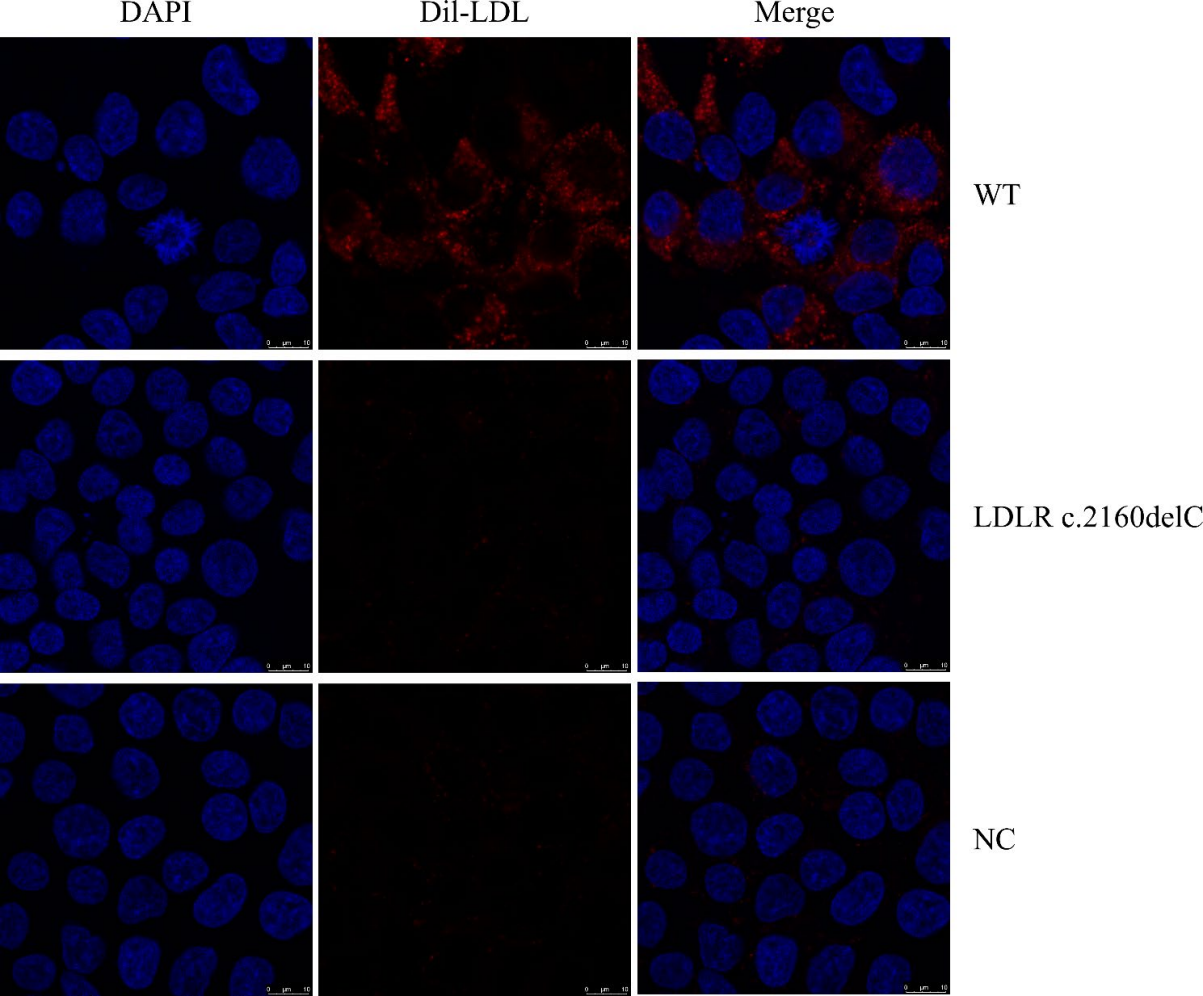
Representative confocal microscopy images. Blue fluorescence represents DAPI, while red fluorescence represents Dil-LDL

To determine the subcellular localization of the LDLR variant, LDLR was incubated with the endoplasmic reticulum marker calnexin antibody as described previously [26]. The results of confocal microscopy showed that the LDLR c.2160delC variant was predominantly trapped in the endoplasmic reticulum (Fig. 4). After incubation in Dil-LDL medium, the uptake of LDL levels in the variant group was significantly reduced compared to those of the WT LDLR group (Fig. 5).

## Discussion

Familial hypercholesterolemia is a common metabolic disorder that follows an autosomal inheritance pattern. Since FH is exposed to high LDL-C levels from birth, it has various hazards to vascular function and is even life-threatening [27]. If untreated, the total cholesterol levels of heterozygous FH can reach 8 to 15 mmol/L, and men and women usually develop coronary heart disease before the ages of 55 and 60, respectively, while homozygous FH has total cholesterol levels of up to 12 to 30 mmol/L, usually developing coronary heart disease early in life and dying before the age of 20 [28, 29]. Nonetheless, FH remains overlooked in many regions and countries [30]. No large-scale epidemiological investigation of FH has been conducted in China, where recognition of this disease remains rudimentary [31]. Therefore, improving the rate of FH diagnosis and exploring its pathogenesis is essential. The present study documents a novel variant of LDLR c.2160delC in a family with FH. By conducting a family investigation and cascade screening, we established that the mode of inheritance for this family lineage is autosomal dominant. Cell biology experiments have also confirmed that LDLR c.2160delC prematurely terminates LDLR transcription. This phenomenon causes the accumulation of proteins in the endoplasmic reticulum without reaching the cell surface to perform their function of clearing LDL. In short, this study confirmed a new pathogenic variant of LDLR, which might provide a more complete pathogenic profile for the diagnosis of FH and lay the foundation for personalized gene therapy for future FH patients.

LDLR is a cell surface glycoprotein primarily responsible for cholesterol uptake from the circulation [32]. After translation, LDLR precursors are folded and modified to mature proteins in the secretory pathway, which are eventually expressed on the plasma membrane of cells [33]. There are 5 functional domains in LDLR, including the anchoring cytoplasmic domain, transmembrane domain, O-linked sugar domain, epidermal growth factor (EGF) precursor homologous domain, and ligand binding domain [34]. Therefore, significant heterogeneity surrounds the mechanisms by which pathogenic mutations in various functional regions cause FH. LDLR mutations can be classified into five types based on their function and synthesis, including type 1 dysfunction of LDLR synthesis (nonsense mutation and splicing mutation); type 2 defect of LDLR trafficking to the cell membrane (generally in ligand-binding domains or EGF precursor homologous domains); type 3 impaired LDL binding (mainly in ligand-binding domain and homologous domain of EGF precursors); type 4 reduced capacity for receptor-mediated endocytosis (mainly in transmembrane domains and cytoplasmic domains); and type 5 diminished LDLR recycling capacity (mainly in homologous domain of EGF precursor) [34, 35]. The new variant reported in the present study is located at LDLR exon 15, corresponding to the O-linked sugar domain [36]. A previous study similarly found that the O-linked sugar domain might regulate LDLR stability or LDLR release from cells [37].

The qPCR results in the present study confirmed that LDLR c.2160delC was a type 1 mutation that could cause premature transcription termination. However, WB confirmed that transcribed sequences could still be translated into defective proteins despite early transcription termination. Flow cytometry and immunofluorescence staining suggested that the defective LDLR protein was trapped in the endoplasmic reticulum and barely reached the cell surface. Based on these experimental results, LDLR c.2160delC is a type 2 mutation. Additionally, the endoplasmic reticulum is the site of the assembly of secreted proteins and membrane proteins, and the retention of LDLR variants in the endoplasmic reticulum induces endoplasmic reticulum stress [38–40]. Activating endoplasmic reticulum stress could further damage cell and tissue function, thus forming a vicious cycle. Therefore, the side effects of deficient LDLR proteins affect not only its own function but also the function of the entire cell.

### Comparisons with other studies and what does the current work add to the existing knowledge

In recent years, the pathogenic gene mutations of FH have gradually become a focus of research. A systematic review of 74 studies on the characteristics of LDLR mutations in the Chinese population showed that LDLR variants were mostly located in the fourth exon, and the main types were LDLR c. G986 A, c. C1747 T, and c.G1879A [41]. Although many LDLR variants have been identified in FH, to my knowledge, this is the first report of the LDLR c.2160delC variant in an FH family. In addition, *in-vitro* experiments have confirmed that the LDLR c.2160delC variant could lead to the loss of LDLR function.

### Study strengths and limitations

This study confirmed that the terminating mutation LDLR c.2160delC could lead to early termination of the transcription process by deleting one base. Moreover, defective protein accumulation in the endoplasmic reticulum might lead to the loss of LDLR function. All the evidence provides clues for the diagnosis and subsequent development of effective treatments in FH. Nonetheless, there are still some limitations in this study. Although the influence of the variant on the total amount of LDLR in cells and the expression of LDLR on the cell surface was detected in this study, the residual activity of LDLR was not detected. The pathogenicity of the LDLR c.2160delC variant has not been demonstrated in vivo. In addition, how to ameliorate the effects of the variant remains unsolved. Therefore, more relevant studies on the in vivo pathological mechanism and treatments are needed to develop more effective treatment methods and improve the prognosis of FH, especially homozygous FH.

## Conclusion

In conclusion, the current study substantiated that the deletion mutation LDLR c.2160delC led to LDLR dysfunction by affecting protein expression and processing. This study contributes to the improvement of the gene mutation spectrum in patients with FH and provides a basis for genetic screening and treatment.

## Electronic supplementary material

Below is the link to the electronic supplementary material.

Supplementary Material 1

Supplementary Material 2

Supplementary Material 3

## Data Availability

The datasets presented in this article are not readily available because sharing of genomic data in the public domain is not allowed according to the requirements of the Institutional Ethics Committee. Requests to access the datasets should be directed to the corresponding authors.
