# Supplementary material for: In vitro assessment of the pathogenicity of the LDLR c.2160delC variant in familial hypercholesterolemia
Source: Lipids Health Dis. 2023 Jun 20;22:77. doi: 10.1186/s12944-023-01848-6 (PMC10280840; doi:10.1186/s12944-023-01848-6)
Supplement: Supplementary file 1 — Supplementary Material 1 [file 12944_2023_1848_MOESM1_ESM.pdf]

Freescience Editorial Team

Certificate of English Editing

Paper Title

The pathogenicity of the LDLR c.2160delC variant in familial hypercholesterolemia

Authors

Shaoyi Lin

This certificate is issued as a confirmation that the paper mentioned above has been proofread and edited for language clarity and grammar by professional editors（Name:William Pat Fong） of our company.

We guarantee that the original message was not distorted, and that the paper is understandable and free of errors assuming that the changes and suggestions given are accepted, and text is not altered without our knowledge

Date of Editing: 03-29-2023
