# Supplementary material for: In vitro assessment of the pathogenicity of the LDLR c.2160delC variant in familial hypercholesterolemia
Source: Lipids Health Dis. 2023 Jun 20;22:77. doi: 10.1186/s12944-023-01848-6 (PMC10280840; doi:10.1186/s12944-023-01848-6)
Supplement: Supplementary file 2 — Supplementary Material 2 [file 12944_2023_1848_MOESM2_ESM.pdf]

20230402231206840043011823792128

WORD COUNT 2600
TIME SUBMITTED 02-APR-2023 11:12PM
PAPER ID 98275092

The pathogenicity of the LDLR c.2160delC variant in familial hypercholesterolemia

Abstract

Background: Familial hypercholesterolemia (FH) is an inherited disorder with markedly elevated low-density lipoprotein cholesterol (LDL-C), and premature atherosclerotic cardiovascular disease. Although many mutations have been reported in FH, only a few have been identified as pathogenic. This study aimed to confirm the pathogenicity of the LDL receptor (LDLR) c.2160delC variant in FH.

Methods: In this study, the proband and her family members were systematically investigated and the pedigree map was drawn. High-throughput whole-exome sequencing was used to explore the variants in this family. Next, quantitative polymerase chain reaction (qPCR), western blot (WB) assays and flow cytometry were conducted to detect the effect of LDLR c.2160delC variant on its expression. The LDL uptake capacity and cell localization of LDLR variants were analyzed by confocal microscopy.

Results: According to DLCN diagnostic criteria, two FH patients were identified with LDLR c.2160delC variant in this family. An *in-silico* analysis suggested that the deletion mutation at the 2160 site of LDLR causes a termination mutation. The results of qPCR and WB verified that the LDLR c.2160delC variant led to early termination of LDLR gene transcription. Furthermore, the LDLR c.2160delC variant caused LDLR to accumulate in the endoplasmic reticulum preventing it from reaching the cell surface and internalizing LDL.

Conclusions: The LDLR c.2160delC variant is a terminating mutation, which plays a pathogenic role in FH.

Keywords: familial hypercholesterolemia, low-density lipoprotein cholesterol, low-density lipoprotein receptor, whole-exome sequencing, endoplasmic reticulum

Introduction

Familial hypercholesterolemia (FH) is a prevalent hereditary metabolic disorder, featured on tendinous or cutaneous xanthomas, extremely low-density lipoprotein cholesterol (LDL-C), arcus corneal, and early onset of cardiovascular disease[1-3]. Current evidence suggests that FH has a prevalence of 1 in 311 among the general population and is more common among atherosclerotic cardiovascular disease (ASCVD) cases[4]. Although significant inroads have been achieved over the past few years, FH remains underdiagnosed and undertreated worldwide[3, 5]. Therefore, strengthening population screening and cascade screening of probands are necessary and effective means to improve the diagnosis rate.

However, treating FH remains a major conundrum for clinicians. Statins, which form the basis of cholesterol-lowering therapy, exhibit significant variations in their ability to lower LDL-C among individuals and bring many adverse effects[6]. Most importantly, the therapeutic effect of statins is below par for FH patients[7]. The PCSK9 inhibitor represents a new class of drugs for treating this patient population. In the latest CREDIT-2 study, tafolecimab was related to a significant decrease in LDL-C levels in Chinese heterozygous FH patients (-57.4% for 150 mg Q2W; -61.9% for 450 mg Q4W)[8]. However, there is an increasing consensus that the lipid-lowering effect of PCSK9 inhibitors for homozygous FH patients is not ideal in adults and children [9-11]. This finding urges us to promptly investigate the pathogenesis of FH and conduct further research for more effective therapies to resolve this conundrum.

It has been established that FH typically arises from genetic mutations that play a vital role in regulating cholesterol balance[12]. LDLR, APOB, PCSK9, and LDLRAP1 are the causative genes identified thus far, with the LDL receptor (LDLR) being the most commonly mutated[1, 13]. More than 4000 LDLR variants have been identified according to the UCL database (http://www.lovd.nl/LDLR)[14, 15]. As a transmembrane receptor mainly expressed in the liver, LDLR can reportedly bind to circulating LDL particles, subsequently internalizing and degrading LDL[12]. An increasing body of literature suggests that mutations at different locations may cause LDLR failure through different mechanisms [16, 17]. Even, two LDLR variants

encoding the same amino acid impair LDLR function differently [18]. Based on these findings, the functionality of the newly identified variants warrants a thorough investigation, providing the theoretical basis for exploring effective FH treatment strategies.

Herein, we sought to explore the causes of FH through family investigation and high-throughput sequencing. Furthermore, cell biology studies were undertaken to identify the pathogenicity of the novel LDLR variant LDLR c.2160delC in this patient population.

Methods and Materials

Study population and samples collection

The proband and her family members were recruited from the First Affiliated Hospital of Ningbo University. All subjects underwent a detailed physical examination and the pedigree was drawn. FH diagnosis was mainly based on the Dutch Lipid Clinic Network (DLCN) criteria. In addition, samples were obtained from all participants for subsequent studies. This study was approved by the Ethics Committee of the First Affiliated Hospital of Ningbo University, and the written informed consent form has been signed by all subjects.

Whole-exome sequencing

Omega Blood DNA Kit (Omega bio-tek, GeorgiaState, USA) was used to isolate genomic DNA from blood samples. The Nano Drop 2000 spectrophotometer (Thermo Fisher Scientific, USA) was used to detect the quality of extracted DNA. The whole-exome sequencing was completed by BGI (Shenzhen, China). First, the original data was processed to generate high-quality clean data, which was then combined with a reference genome (GRCh37/hg19) to obtain the original alignment results in the bam file. Finally, small fragment insertion/deletion and single nucleotide variants were recognized.

Sanger sequencing

The validation of Next-generation sequencing (NGS) results was done by Sanger sequencing. PCRs were performed in 50μL of a mixture containing 25μl 2XES Taq

Master Mix (CW0690H, NYbio, China), 2μl primer (F: 5'-GTCATCTTCCTTGCTGCCTGTTTAG-3', R: 5'-GTTTCCACAAGGAGGTTTCAAGGT-3'), 500ng DNA, and enzyme-free water (supplemented to 50μl). Amplification was performed on Mastercycler® nexus X2 (Eppendorf, Germany) with the following parameters: 94°C 2min; 40 cycles: 94°C 30s, 59°C 30s, 72°C 30s; 72 2min. The amplified products were sent to BGI for Sanger sequencing and Chromas software was applied to analyze the sequencing results.

In silico analysis

The pathogenic potential of gene variants was analyzed by MutationTaster (http://www.mutationtaster.org/)[19]. For LDLR, the reference genome NM_000527.4 was used.

Generation of mutant expression constructs

Plasmid was synthesized by GENECHEM (Shanghai, China). All variants described in this study were based on the encoded LDLR sequence (NM_000527.4). GFP was added to the vector to assess the efficiency of plasmid transfection in living cells. Site-directed mutagenesis was applied to introduce 2160delC mutation into the LDLR expression vector.

Cell culture and transfection

A culture of human embryonic kidney cells (HEK-293T) was performed in DMEM (Cytiva, China) supplemented with 100 U/ml penicillin/streptomycin (Beyotime, China) and 10% FBS (Pan, China). After HEK-293T cells had reached 80% confluence, plasmid transfection was performed 12 using Lipofectamine 2000 Reagent (Invitrogen, USA).

Quantitative real-time PCR

Forty-eight hours after transfection, RNA-Solv® Reagent (Omega,USA) was used for total RNA extraction. 13 Reverse transcription was performed using the HiScript cDNA Synthesis Kit (CW2569M, CWBIO, China), which was performed on Mastercycler® nexus X2 (Eppendorf, Germany) with the following parameters: 42°C 50min; 85°C 5min. This study was conducted using FastStart Essential DNA Green Master (069242204001, Roche, Switzerland) and LightCycler®480 (Roche,

Switzerland) for quantitative real-time PCR. The primer for LDLR was F: 5'-CCTGACTCCGCTTCTTCTGCCCCAG-3', R: 5'-ACGCAGAAACAAGGCGTGTGCCAC-3'. The primer for GAPDH was F: 5'-GGAGTCAACGGATTTGGT-3', R: 5'-GTGATGGGATTTCCATTGAT-3'. LDLR variants expression was measured using the $2^{-\Delta\Delta Ct}$ method.

Western blot

RAPI buffer (Solarbio, China) containing protease and phosphatase inhibitors was used to lyse the samples. The cellular lysates were added to 1× loading buffer (Beyotime, China), which were run on 8% SDS-PAGE gradient gel at 120 V for 90min. Then, the proteins were transferred onto PVDF membranes (Merck, Germany). After blocking, the primary antibodies monoclonal mouse anti-LDLR (1:1000, ab204941, Abcam, UK) and monoclonal mouse anti-GAPDH (1:5000, AC033, ABclonal, China) were used in this study. The proteins were visualized using a fully automated chemiluminescence/fluorescence image analysis system (Tanon, China), and the protein integration density was quantified using ImageJ software.

Flow Cytometry

After transfection, cells were collected into 1.5ml EP tubes (0.5-1× 10^6 per tube). Then, HEK-293T cells were blocked and cultured with anti-human LDLR monoclonal antibody conjugated with APC (5μl/test, ab275614, Abcam, UK) for 30 min at RT. Fluorescence intensity was measured on a Beckman CytoFlex S flow cytometer (Beckman Coultern, China) using the filter FL3 (excitation: 645 nm, emission: 660 nm).

Immunofluorescent staining

For immunostaining, HEK-293T cells were fixed for 10 minutes in 4% paraformaldehyde. Cells were blocked in 5% donkey serum diluted with 0.1% TBST, then incubated with mouse anti-LDLR antibody (1:800, ab204941, Abcam, UK) and rabbit anti-calnexin antibody (1:100, P27824, CST, USA) for 12 hours at 4°C. After incubation with the respective secondary antibodies, confocal microscopy was used to determine whether the protein was trapped in the endoplasmic reticulum.

Additionally, the uptake capacity of LDLR variants was analyzed by immunocytochemistry. After a confluence of 50% was reached, the medium was

changed to DMEM with 0.3% FBS. Twelve hours later, the cells were incubated at 37°C for a further 4 hours with 20 μg/ml Dil-LDL (Thermo Fisher Scientific, USA). After 4% paraformaldehyde fixation, HEK-293T cells on coverslips were stained with DAPI and visualized with confocal microscopy.

Statistical Analysis

The statistical analyses were carried out using SPSS 25.0 software (SPSS Inc., Chicago, USA). It was statistically significant when the P-value < 0.05 in the two-tailed analysis.

Results

Clinical characteristics of the proband and her family

The proband was a 33-year-old female enrolled at The First Affiliated Hospital of Ningbo University for dyslipidemia and corneal arch. At baseline, the total cholesterol was 11.26 mmol/L and LDL-C was 8.11 mmol/L. The patient's family history revealed that her father, brother, and daughter also had dyslipidemia (Figure 1). According to the DLCN diagnostic criteria, the proband and her father and daughter were suspected of FH (Table 1).

Pathogenic variants and *in-silico* analysis

Through whole-exon sequencing (II-2, II-3, III-1) and bioinformatics analysis, a potential pathogenic mutation was identified in this family, located at the 2160 site of LDLR (Figure 2A). Sanger sequencing further confirmed the existence of LDLRc.2160delC in the corresponding patients (Figure 2B). MutationTaster software was used to predict the pathogenicity of the novel LDLR variant LDLR c.2160delC in FH. LDLR c.2160delC variant represented a potential pathogenic variant, which could lead to early termination of mRNA transcription.

LDLR c.2160delC variant affected the expression of LDLR

Next, qPCR, WB, and flow cytometry assays were conducted to validate that LDLR c.2160delC could induce premature transcription termination. Based on the mutation site 2160 of the gene sequence, PCR primers were designed, and LDLR mRNA expression levels were extremely low compared to WT (Figure 3A, $P < 0.01$).

LDLR protein expression in each group was analyzed by western blot (Figure 3B). In the group expressing WT LDLR, the precursor form is 120 kDa, and the mature form is 150 kDa. Since LDLR c.2160delC causes premature termination, the protein's size was smaller than normal LDLR (< 120 kDa). Moreover, neither precursors nor mature forms were observed. Besides, the LDLR on the cell surface was further analyzed by flow cytometry (Figure 3). There was almost no LDLR on the surface in the group of LDLR c.2160delC. These results indicated that LDLR c.2160delC was a terminating mutation that could prevent LDLR transport to the cell membrane.

LDLR c.2160delC variant was accumulated in the endoplasmic reticulum and reduced Dil-LDL uptake in HEK-293T cells

To determine the subcellular localization of the LDLR variant, LDLR was incubated with the endoplasmic reticulum marker calnexin antibody as described previously[20]. During confocal microscopy, the LDLR c.2160delC variant was predominantly trapped in the endoplasmic reticulum (Figure 4). After incubated in Dil-LDL medium, the LDL levels of the variant group was significantly reduced compared to WT LDLR (Figure 5).

Discussion

Familial hypercholesterolemia is a common metabolic disorder that follows an autosomal inheritance pattern. Since FH is exposed to high LDLC levels from birth, it has various hazards to vascular function and is even life-threatening[21]. Nonetheless, FH remains overlooked in many regions and countries [22]. To our knowledge, no large-scale epidemiological investigation of FH has been conducted in China, where recognition of this disease remains rudimentary[23]. Therefore, improving the rate of FH diagnosis and exploring its pathogenesis is essential. The present study documents a novel variant of LDLR c.2160delC in a family with FH. By conducting a family investigation and cascade screening, we established that the mode of inheritance for this family lineage is autosomal dominant. Cell biology experiments have also confirmed that LDLR c.2160delC could prematurely terminate LDLR transcription. This phenomenon causes the accumulation of proteins in the endoplasmic reticulum

without reaching the cell surface to perform their function of clearing LDL.

LDLR is a cell surface glycoprotein primarily responsible for cholesterol uptake by peripheral cells from the circulation[24]. After translation, LDLR precursors are folded and modified to mature proteins in the secretory pathway, which are eventually expressed on the plasma membrane of cells[25]. There are 5 functional domains in LDLR, including the anchoring cytoplasmic domain, transmembrane domain, O-linked sugar domain, epidermal growth factor (EGF) precursor homologous domain, and ligand binding domain[26]. Therefore, significant heterogeneity surrounds the mechanisms by which pathogenic mutations in various functional regions cause FH. LDLR mutations can be classified into five types based on their function and synthesis, including type 1 dysfunction of LDLR synthesis, type 2 defect of LDLR trafficking to the cell membrane, type 3 impaired LDL binding, type 4 reduced capacity for receptor-mediated endocytosis, type 5 diminished LDLR recycling capacity [27]. The new variant reported in the present study is located at LDLR's exon 15, corresponding to the O-linked sugar domain[28]. A previous study similarly found that O-linked sugar domain might regulate LDLR stability or LDLR release from within cells[29].

The qPCR results in the present study confirmed that LDLR c.2160delC was a type 1 mutation that could cause premature transcription termination. However, WB confirmed that transcribed sequences could still be translated into defective proteins despite early transcription termination. Flow cytometry and immunofluorescent staining suggested that the defective LDLR protein was trapped in the endoplasmic reticulum and could barely reach the cell surface. Based on these experimental results, it is highly conceivable that LDLR c.2160delC is a type 2 mutation. Additionally, the endoplasmic reticulum is the site of the assembly of secreted proteins and membrane proteins and the retention of LDLR variants in the endoplasmic reticulum induced endoplasmic reticulum stress[30-32]. Activating endoplasmic reticulum stress could further damage cell and tissue function, thus forming a vicious circle. Therefore, further exploration is warranted to identify the side effects of deficient LDLR proteins.

Comparisons with other studies and what does the current work add to the

existing knowledge

In recent years, the pathogenic gene mutations of FH have gradually become a focus of research. A systematic review of 74 studies on the characteristics of LDLR mutations in Chinese population showed that LDLR variants were mostly located in the fourth exon, and the main types were LDLR c.G986 A, c.C1747 T, and c.G1879A[33]. Though many LDLR variants have been identified in FH, to my knowledge, this is the first report of the LDLR c.2160delC variant in an FH family. In addition, *in-vitro* experiments have confirmed that the LDLR c.2160delC variant could lead to the loss of LDLR function.

Study strengths and limitations

This study confirmed that the terminating mutation LDLR c.2160delC could lead to early termination of transcripion process by deleting one base. Moreover, the defective protein accumulation in the endoplasmic reticulum might lead to the loss of LDLR function. All the evidence provides clues for the diagnosis and subsequent development of effective treatments in FH. Nonetheless, there are still some limitations in this study. The pathogenicity of the LDLR c.2160delC variant has not been demonstrated in vivo. In addition, how to ameliorate the effects of the variant remains unsolved. Therefore, more relevant studies on the *in vivo* pathological mechanism and treatments are needed to develop more effective treatment methods and improve the prognosis of FH, especially homozygous FH.

Conclusion

In conclusion, the current study substantiated that the deletion mutation LDLR c.2160delC led to LDLR dysfunction by affecting protein expression and processing. This study contributes to the improvement of gene mutation spectrum in patients with FH, and also provides a basis for genetic screening.

20230402231206840043011823792128

ORIGINALITY REPORT

10%

SIMILARITY INDEX

PRIMARY SOURCES

1	www.ncbi.nlm.nih.gov<br>Internet	60 words — 2%
2	www.frontiersin.org<br>Internet	37 words — 1%
3	www.researchgate.net<br>Internet	31 words — 1%
4	pure.uva.nl<br>Internet	26 words — 1%
5	Marianne Abifadel, Catherine Boileau. "Genetic and molecular architecture of familial hypercholesterolemia", Journal of Internal Medicine, 2022<br>Crossref	18 words — 1%
6	Tong-Mei Yang, Miao Miao, Wen-Qian Yu, Xue Wang, Fang-Jie Xia, Yan-Jie Li, Shou-Dong Guo. "Targeting macrophages in atherosclerosis using nanocarriers loaded with liver X receptor agonists: A narrow review", Frontiers in Molecular Biosciences, 2023<br>Crossref	18 words — 1%
7	"Lipoprotein Receptor Signaling", Encyclopedia of Hormones, 2003<br>Crossref	16 words — 1%

8	Yi Chen, Wei Chen, Yiming Ren, Shuling Li et al. "Lipid nanoparticle-encapsulated VEGFa siRNA facilitates cartilage formation by suppressing angiogenesis", International Journal of Biological Macromolecules, 2022<br>Crossref	16 words — 1%
9	Xudong Wang, Yanshi Li, Min Pan, Tao Lu, Min Wang, Zhihai Wang, Chuan Liu, Guohua Hu. "CEACAM5 inhibits the lymphatic metastasis of head and neck squamous cell carcinoma by regulating epithelial–mesenchymal transition via inhibiting MDM2", Clinical Science, 2022<br>Crossref	15 words — 1%
10	Hao Jiang, Huihui Zhou, Chengdong Liu, Xuan Wang, Kangsen Mai, Gen He. " Effects of phosphatidic acid on growth and antioxidant capacity in juvenile turbot, L., fed with high plant protein ‐ based diets ", Journal of the World Aquaculture Society, 2021<br>Crossref	14 words — 1%
11	Yong Zhang, Yanfen Niu, Qiuyan Weng. "Ginkgetin promotes proliferation and migration of Schwann cells via PIGF/p38 MAPK signaling pathway", Tissue and Cell, 2022<br>Crossref	14 words — 1%
12	medsci.org<br>Internet	10 words — < 1%
13	abccardiol.org<br>Internet	8 words — < 1%

EXCLUDE QUOTES	OFF	EXCLUDE SOURCES	OFF
EXCLUDE BIBLIOGRAPHY	ON	EXCLUDE MATCHES	OFF
