# Supplementary material for: In vitro assessment of the pathogenicity of the LDLR c.2160delC variant in familial hypercholesterolemia
Source: Lipids Health Dis. 2023 Jun 20;22:77. doi: 10.1186/s12944-023-01848-6 (PMC10280840; doi:10.1186/s12944-023-01848-6)
Supplement: Supplementary file 3 — Supplementary Material 3 [file 12944_2023_1848_MOESM3_ESM.pptx]

## Slide 1
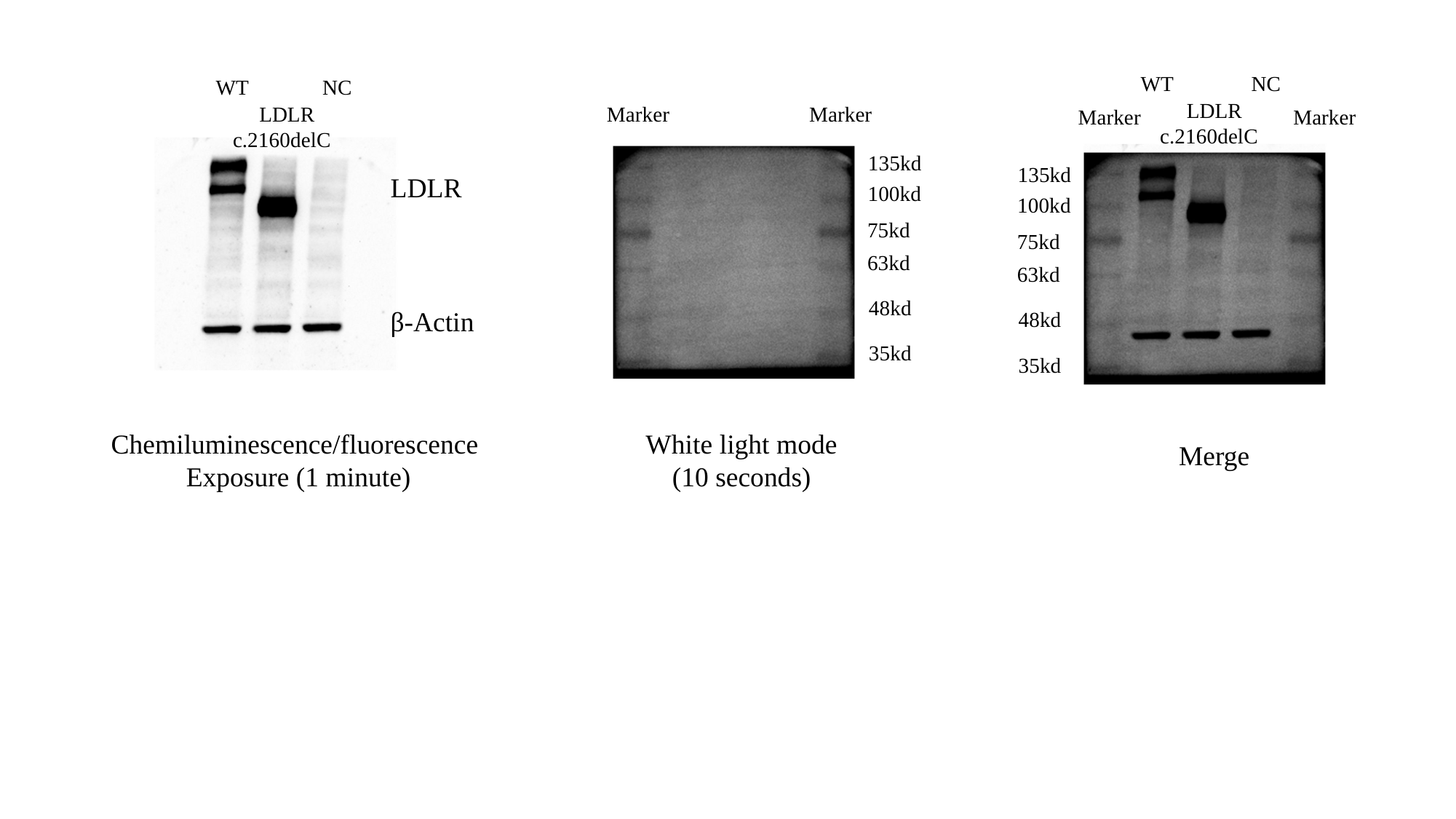

WT
NC
WT
NC
LDLR
 c.2160delC
Marker
LDLR
 c.2160delC
Marker
Marker
Marker
135kd
135kd
LDLR
100kd
100kd
75kd
75kd
63kd
63kd
48kd
β-Actin
48kd
35kd
35kd
White light mode
(10 seconds)
Chemiluminescence/fluorescence
Exposure (1 minute)
Merge
